# Non-blind acoustic invisibility by dual layers of homogeneous single-negative media

**DOI:** 10.1038/srep42533

**Published:** 2017-02-14

**Authors:** He Gao, Yi-fan Zhu, Xu-dong Fan, Bin Liang, Jing Yang, Jian-Chun Cheng

**Affiliations:** 1 Collaborative Innovation Center of Advanced Microstructures and Key Laboratory of Modern Acoustics, MOE, Institute of Acoustics, Department of Physics, Nanjing University, Nanjing 210093, P. R. China

## Abstract

Non-blind invisibility cloaks allowing the concealed object to sense the outside world have great application potentials such as in high-precision sensing or underwater camouflage. However the existing designs based on coordinate transformation techniques need complicated spatially-varying negative index or intricate multi-layered configurations, substantially increasing the difficulty in practical realization. Here we report on the non-blind acoustic invisibility for a circular object in free space with simple distribution of cloak parameters. The mechanism is that, instead of utilizing the transformation acoustics technique, we develop the analytical formulae for fast prediction of the scattering from the object and then use an evolutionary optimization to retrieve the desired cloak parameters for minimizing the scattered field. In this way, it is proven possible to break through the fundamental limit of complementary condition that must be satisfied by the effective parameters of the components in transformation acoustics-based cloaks. Numerical results show that the resulting cloak produces a non-bflind invisibility as perfect as in previous designs, but only needs two layers with homogenous single-negative parameters. With full simplification in parameter distribution and broken symmetry in complementary relationship, our scheme opens new route to free-space non-blind invisibility, taking a significant step towards real-world application of cloaking devices.

During the last decade, considerable attentions have been and continue to be dedicated to acoustic metamaterials that enable control over acoustic wave in unprecedented ways^1^,^2^,^3^,^4^,^5^,^6^,^7^,^8^,^9^,^10^,^11^,^12^,^13^,^14^. One of the fanciest metamaterial-based devices is the “invisibility cloak” with potential applications in a wide range of scenarios. The scheme of cloaking is first presented for electromagnetic waves^1^,^2^, which provides perfect cloaking effects but prevents the cloaked object from interacting with the environment. To address this problem, Lai *et al*. have come up with the scheme of “external cloaking” which requires double-negative materials with parameters dependent of the cloaked object that are difficult to achieve in practice^14^. Later Zhu *et al*. have proposed the idea of “superlens cloak” with complementary media made of single-negative materials, which allows the cloaked object to share information with the outside world^4^. Although the usage of single-negative media already represents a significant simplification in comparison with those designs containing double-negative media, the existing non-blind cloaks, built via transformation acoustics technique, still need to be constructed by either two layers of single-negative media with parameters dependent of spatial position or by a great number of layers with homogeneous single-negative media^4^,^5^. These requirements are challenging for the experimental realization and practical application of non-blind cloaking devices. Despite the potential of acoustic metamaterials to serve as negative index media, the metamaterial implementation of continuous spatial modulation of negative parameters is difficult to achieve in practice. On the other hand, the effective medium approximation may become invalid in each individual thin layer serving as the building block of a multi-layer structure that needs to contain enormous layers to produce a satisfying cloaking effect^5^. It would therefore be of both fundamental and practical significance to pursue an alternative scheme exempted from spatially-varying parameters and large layer number.

In this article, we propose to produce non-blind acoustic invisibility by using a dual layer structure filled with two homogeneous single-negative media, breaking through the limit in the transformation acoustics-based designs. Based on an inherently different scheme that straightforwardly realize a perfect impedance match between an object and the surrounding medium, the proposed scheme enables the ingredients of cloak not to strictly satisfy the complementary condition indispensable for the existing approaches. By developing the analytical formulae to fast calculate the scattering strength from a circular object and then employing genetic algorithm as an efficient method to achieve the optimal configuration for maximal scattering cancellation. As a result, the designed cloak only needs to consist of a negative mass density and a negative bulk modulus media with judiciously designed parameters, which represents the simplest configuration ensuring the fundamental requirement of evanescent wave restoration^15^,^16^,^17^. The performance of the resulting cloak is demonstrated numerically via production of non-blind invisibility effect that allows the cloaked object to receive the incident signal undistortedly while suppressing its scattered field to near-zero. Quantitative evaluation on the cancellation of scattering field further reveals that in comparison with a transformation acoustics-based non-blind cloak with the same thickness and parameter complexity, our designed cloaks give rise to an invisibility phenomenon with equivalent efficiency despite the full simplification in its parameter distribution. We have also demonstrated the robustness of the cloaking effect against the deviation of the structural parameters of the cloak, which helps to substantially facilitate the fabrication and application of the non-blind cloak considering the avoidable difficulty in exactly achieving the predicted negative parameters in practice.

## Results

**Figure 1:**
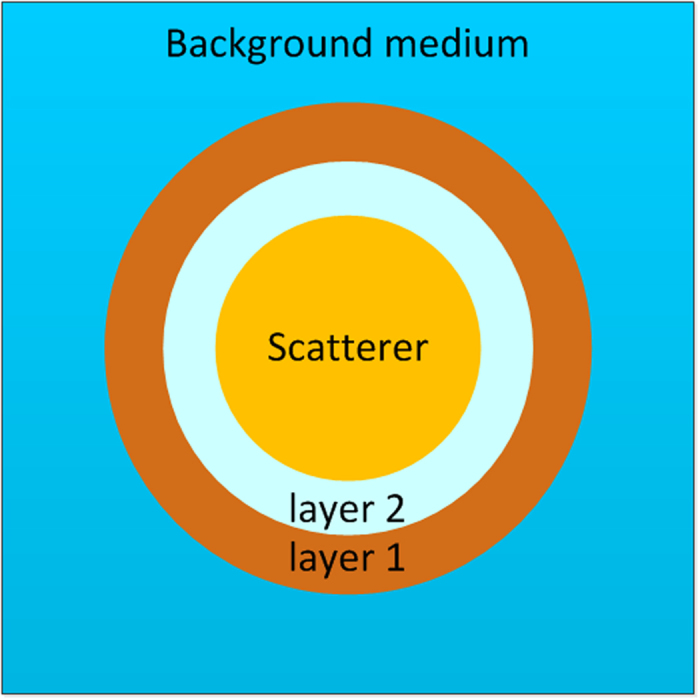
Schematic of our theoretical model. The innermost cylinder is the scatterer to be cloaked, the cloak is composed of layers 1 and 2 whose structural parameters needs to be designed.

Our aim is to design a dual-layer cloak comprising two media with different homogeneous single-negative parameters, which would be later proven the simplest possible structure for building an non-blind cloak that makes an object inside it to receive incident signals but to generate no scattered wave, as schematically depicted in Fig. 1. For simplicity without losing generality, we consider a two-dimensional (2D) case with the cloaked object chosen as a circular scatterer. For better evaluation of the performance of our proposed cloak via a comparison with the previous transformation acoustics-based designs, as will be demonstrated later, we use a scatterer with parameters identical with those used in ref. 5: modulus *κ*_s_ = 4*κ*_0_/9, mass density *ρ*_s_ = *ρ*_0_ and radius *a* = 1 cm with *κ*_0_ = 21.9 GPa and *ρ*_0_ = 998 kg/m^3^ being the modulus and mass density of the background medium (chosen as water) respectively. Moreover, the total thickness of the cloak is 0.5 cm which is the same with the ten-layer cloak model presented there^5^.

From the viewpoint of coordinate transformation, the annular region occupied by layers 1 and 2 in Fig. 1 is a “hole” that needs to be filled with pairs of single-negative media strictly satisfying the complementary conditions to ensure the vanishing of this region in the virtual space. This leads to the aforementioned requirements of spatially-varying parameters or complicated multi-layered structures, making the simple cloak depicted in Fig. 1 unattainable with the existing schemes. Here we do not need to rely on the complementary condition that is crucial for the success of the designs of transformation acoustics-based cloaks. Instead, we begin from the basic problem of how the incident plane wave will be scattered by the object wrapped with the designed cloak. Based on this, we make an attempt to suppress the generated scattered field with an inverse design scheme, which would enable a full simplification in the cloak structure without sacrificing the efficiency of invisibility. Hence we first give an analytical analysis to the acoustic field of the scatterer and derive the formulae for predicting the performance of the designed cloak quantitatively.

Consider the scattered wave field generated by a three-layer structure shown in Fig. 1 when illuminated by the incident plane wave 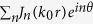. The wave field at the position (*r, θ*) in the surrounding medium is represented as a sum of incident wave field 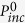 and scattered wave field 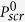, which can be expressed as^18^


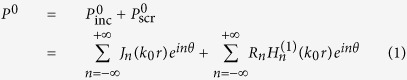


where *k*_0_ is the wave number in the surrounding medium, *R*_*n*_ represents the scattering coefficient, *J*_*n*_ is *n*-order Bessel function and 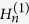 is *n*-order Hankel function of the first kind. The wave in each layer can be represented by a sum of standing wave field 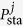 and radiation wave field 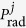:


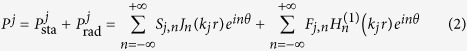


where the subscripts *j* = 1, 2 refers to layers 1 and 2 respectively, *S*_*j*_,_*n*_ and *F*_*j,n*_ are determined by the standing wave and the radiation wave respectively. In the inner scatterer, there only exists standing wave pressure field, expressed as


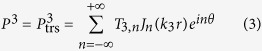


where *T*_3, *n*_ is determined by the standing wave. Then the parameters of *M*_*j, n*_ and *R*_*j, n*_ can be expressed as


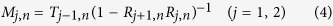



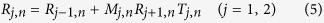


The parameters of *S*_*j, n*_ and *F*_*j, n*_ describing respectively the standing wave and the radiation wave for each layer can be formulated as


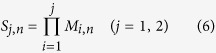



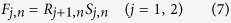



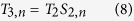


From Eqs (4, 5, 6, 7, 8) we can obtain the values of *R*_*n*_, *S*_*j, n*_, *F*_*j, n*_ (*j* = 1, 2), *T*_3,*n*_ to get the acoustic field of the entire space.

According to the asymptotic expression of Hankel function at infinity, the scattering pressure field for the multilayer circular scatterer can be expressed as


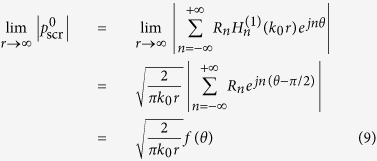


where 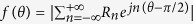 is defined as the scattering form factor which is effective in describing the scattering pattern^19^. For quantitatively evaluating the performance of the resulting devices, we introduce the parameter of total scattering cross section *σ*, defined as^19^


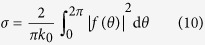


Obviously the total scattering cross section *σ* of an acoustic field can be used as a criterion for estimating unambiguously and precisely how well the object becomes acoustically invisible in the presence of our designed cloak. The optimal performance is achieved by adjusting the structural parameters to yield a total scattering cross section as close to zero as possible. For this purpose, the structural parameters in this system add up to five: the ratio *τ* between the thicknesses of layers 1 and 2, the densities *ρ*_1_, *ρ*_2_ and the bulk moduli *κ*_1_, *κ*_2_ of these two cloak layers. Mathematically, we aim to seek the minimum of a function with five input parameters, composed of the above structural parameters of the cloak, and with one output chosen as the absolute value of the total scattering cross section *σ*. Next we will accomplish the design of non-blind invisibility cloak with evolutionary optimization method.

Here a genetic algorithm (GA) is used to find the optimal design parameters for generating the minimized scattered field at the desired frequency^20^. Based on Charles Darwin’s theory of evolution^21^,^22^, the genetic algorithm is an effective optimization method to search for the optimal solution by simulating the process of natural selection. In a genetic algorithm, once the approximate ranges of the parameters are known, the possible solutions can be encoded by a number of binary genes. The best fit genes would be passed on from one generation to the next. After the process of repeated ranking, selection, crossover, mutation in a number of iterations, and the evaluation by the objective fitness function, the global minimum in the search space can be found.

To apply the genetic algorithm, we employ the absolute value of the total scattering cross section *σ* to serve as the fitness function. Each input parameter is encoded as binary chromosome, which is associated with a value of the fitness (*σ*). Through adjusting these input parameters, the genetic algorithm finds the global optimal value of the objective function, which corresponds to a maximal reduction of the acoustic scattering from the cloaked object.

**Figure 2:**
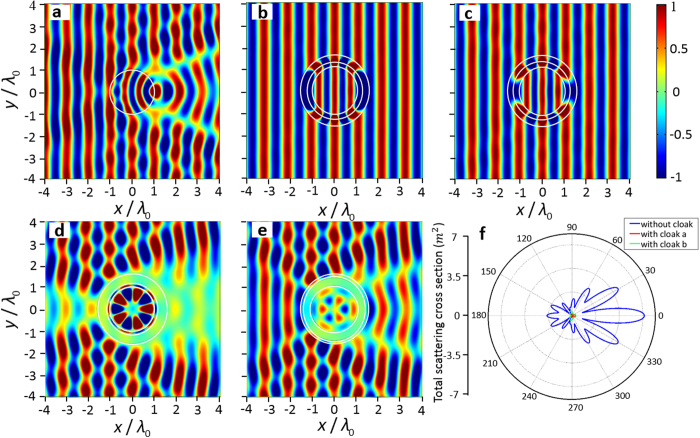
Performance of the designed non-blind invisibility cloak. (**a**) Acoustic field distribution of the bare circular scatterer without cloak. (**b**–**e**) Acoustic field distributions of the circular scatterer with optimized two-layer cloaks of material properties in case (**a**) (*ρ*_1_ < 0, *κ*_1_ > 0, *ρ*_2_ < 0, *κ*_2_ < 0), case (**b**) (*ρ*_1_ > 0, *κ*_1_ < 0, *ρ*_2_ < 0, *κ*_2_ > 0), case (**c**) (*ρ*_1_ < 0, *κ*_1_ > 0, *ρ*_2_ < 0, *κ*_2_ > 0) and case (**d**) (*ρ*_1_ > 0, *κ*_1_ < 0, *ρ*_2_ > 0, *κ*_2_ < 0), subject to plane wave incidence. (**f**) Total scattering cross sections of the circular scatterer for three different cases: the bare circular scatterer, and the same scatterer protected by the dual layers cloak in cases (**a**,**b**).

During the optimization procedure, in order to have the equivalent parameter complexity with Xu’s model^5^, the bulk moduli and mass densities of the two-layer cloak are chosen within the range of −2*κ*_0_ to 2*κ*_0_ and −2*ρ*_0_ to 2*ρ*_0_, respectively, and the thicknesses of the two layers are *t*_1_ = 0.5*τ*/(1 + *τ*) and *t*_2_ = 0.5*τ*/(1 + *τ*). Here we assume that both two layers are composed of homogeneous single-negative materials, which means the density *ρ* and bulk modulus *κ* in each layer must satisfy the inequality *κ* · *ρ* < 0. Then there would be four possible cases: (a) *ρ*_1_ < 0, *κ*_1_ > 0, *ρ*_2_ > 0, *κ*_2_ < 0, (b) *ρ*_1_ > 0, *κ*_1_ < 0, *ρ*_2_ < 0, *κ*_2_ > 0, (c) *ρ*_1_ < 0, *κ*_1_ > 0, *ρ*_2_ < 0, *κ*_2_ > 0, and (d) *ρ*_1_ > 0, *κ*_1_ < 0, *ρ*_2_ > 0, *κ*_2_ < 0. In addition, the perfect restoration of information carried by the incident wave needs the two layers to support the attenuation and amplification of evanescent wave respectively, which means the mass densities of the two layers need to be oppositely signed^15^,^16^. It is therefore expectable that in cases (c) and (d) we would not be able to satisfy such a requirement for rebuilding the incident wave in the cloaked region, which has been verified via numerical simulations^16^,^17^, as is shown in Fig. 2(d) and (e). As a consequence, cases (a) and (b) are the only cases in which the designed structures can be expected to exhibit the potential to yield the desired cloaking effect via parameters optimization.

Figure 2(a) shows the acoustic field distribution of the bare scatterer without cloak impinged by an incident plane wave at frequency of 1.48 × 10^5^ Hz, in which the acoustic field is strongly disturbed by the scatterer. The simulated acoustic field generated by the scatterer wrapped by the designed cloak with parameters optimized for the aforementioned cases (a) and (b) are displayed in Fig. 2(b) and (c) respectively. The optimized material properties of the cloaking media are *τ* = 1.27, *ρ*_1_ = −1.45 *ρ*_0_, *κ*_1_ = 1.95 *κ*_0_, *ρ*_2_ = 1.46 *ρ*_0_ and *κ*_2_ = −1.32 *κ*_0_ for Fig. 2(b), and *τ* = 1.17, *ρ*_1_ = 1.09 *ρ*_0_, *κ*_1_ = −0.82 *κ*_0_, *ρ*_2_ = −1.12 *ρ*_0_ and *κ*_2_ = 0.50 *κ*_0_ for Fig. 2(c). It is apparent that the optimized parameters of layers 1 and 2 are oppositely signed, i.e., *ρ*_1_*ρ*_2_ < 0 and *κ*_1_*κ*_2_ < 0, but do not satisfy the complementary condition. Comparison between the results shown in Fig. 2(a) and in Fig. 2(b) and (c) clearly demonstrate that the scattered fields have been substantially suppressed by the presence of cloak while the incident wave transmits into the cloaked region with no distortion in the wave front, verifying the effectiveness of our non-blind cloak designed with evolutionary optimization. We have also proved that such non-blind invisibility effect corresponds to a nearly perfect impedance match at the outer surface of the designed cloak, which is validated by inspecting the effective acoustic impedance of the whole three-layer structure that is calculated to be approximately equal to the impedance of the surrounding medium. To better evaluate the concealment effects of the optimized two-layer cloaks in a quantitative manner, we have further calculated the total scattering cross sections of the structures in Fig. 2(a–c) respectively, and plotted the results in Fig. 2(f). The amplitudes of the total scattering cross sections are reduced by one order of magnitude when the bare scatterer is wrapped by the optimized dual-layered structures. In addition, the total scattering cross section *σ* defined in Eq. (4) is calculated for each acoustic pressure field. The results prove that the total cross sections in cases (a) and (b) are almost the same as the total cross section of the ten-layer cloak in Xu’s model^5^.

**Figure 3:**
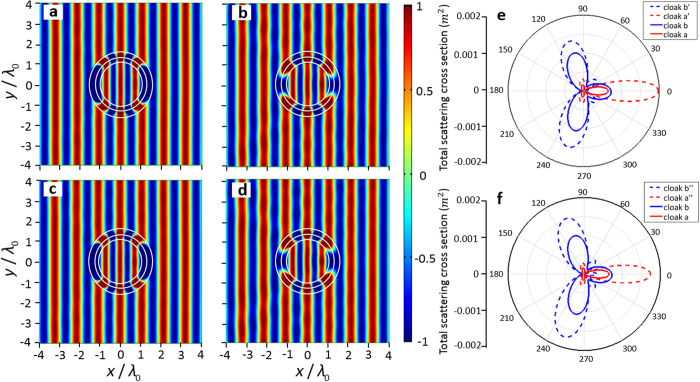
Robustness of the cloaking effect against the deviation of the material parameters of the cloak. (**a**,**b**) Acoustic field distributions for the cases in which the structure parameters are identical same with cases (**a**,**b**) in Fig. 2, except that the mass densities for layers 1 and 2 of the dual-layered structure are set to be 0.5 *ρ*_*1*_ and 0.5 *ρ*_2_ respectively. (**c**,**d**) Acoustic field distributions for the cases in which the structure parameters are identical with cases (**a**,**b**) in Fig. 2, except that the moduli for layers 1 and 2 of the dual-layered structure are set to be 1.5 *κ*_1_ and 1.5 *κ*_2_ respectively. (**e**) Total scattering cross sections of the same scatterer protected by four different cloaks: cloak in case (**a**) (red line), cloak in case (**b**) (blue line), cloak in (**a**) (red dashed line) and cloak in (**b**) (blue dashed line). (**f**) Total scattering cross sections of the same scatterer protected by four different cloaks: cloak in case (**a**) (red line), cloak in case (**b**) (blue line), cloak in (**c**) (red dashed line) and cloak in (**d**) (blue dashed line).

In practical implementation of the designed cloak by metamaterials, it would not be easy to achieve the desired negative parameters perfectly which has to depend on elaborately tuned resonant effects^23^,^24^. Hence it is necessary to investigate how the performance of the resulting devices will be affected if the cloaked media do not have exactly the same effective parameters as given by our evolutionary optimization. Figure 3 shows the typical results of the total acoustic field when the mass densities and bulk moduli of optimized cloak are manually adjusted to deviate notably from their respective original values. In the two particular cases shown in Fig. 3(a) and (b), the mass densities of the two layers of the cloak are chosen as 0.5 *ρ*_1_ and 0.5 *ρ*_2_ respectively while the other structural parameters are the same as those in the aforementioned cases (a) and (b). On the other hand, the results of another two cases in which the moduli of the dual-layer cloak are chosen as 1.5 *κ*_1_ and 1.5 *κ*_2_ with other parameters unchanged are shown in Fig. 3(c) and (d) respectively. To better observe the distortion from the optimal invisibility, we have further calculated the total scattering cross sections of the structures in Figs 2(b,c) and 3(a–d) respectively as is shown in Fig. 3(e) and (f). The numerical results shown in Fig. 3 clearly demonstrate that the dual-layer cloaks keep nearly perfect non-blind invisibility effect despite the change of the densities and moduli in a relatively broad range. When the bulk moduli of the dual-layer cloaks range from 0.7 *κ*_opt_ to 2.5 *κ*_opt_ with *κ*_opt_ being the optimized moduli and mass densities of the dual-layer cloaks range from 0.2 *ρ*_opt_ to 1.5 *ρ*_opt_ with *ρ*_opt_ being the optimized densities, the cloaks in cases (a) and (b) always possess nearly perfect invisibility effect. Such robustness of the cloaking performance to the deviation of the effective parameters of the cloak, which should be helpful to significantly facilitate the practical fabrication and application of our designed non-blind cloaks. Our designed non-blind cloaks should thus be able to work at different frequencies as long as the desired negative parameters can be achieved accurately, which requires each individual unit cells of metamaterial to be much smaller than the working wavelength in their practical implementation. For metamaterials implemented by specific resonant units, the negative parameters usually change very fast in the vicinity of resonances. But the validity of our proposed scheme is not restricted by the practical implementation of negative index media and should be able to stand in a broad band if non-resonant structure can be explored for producing non-dispersive negative effective parameters.

## Discussion

It is of great scientific significance and application potentials to pursue an acoustic cloaking structure exempted from spatially-varying parameters and large layer number in acoustic field. We have presented a dual-layer acoustic cloak made up of homogeneous single-negative medium in this work, which cancels the scattered field from a circular object. Instead of using the transformation acoustics technique to mathematically derive the desired parameters that are proven to strictly satisfy the complementary condition, here we propose an essentially different scheme of producing non-blind invisibility by straightforwardly eliminating the impedance mismatch responsible for the scattering effect to guide the incident wave into the cloaked region reflectionlessly. We give analytical analysis on the scattering by multilayer concentric circular scatterers and derive the analytical solutions of the scattered field. Based on this, a genetic algorithm is used to obtain the optimized material properties for the dual-layer cloak which ensure global minimum for the scattered field. We demonstrate the effectiveness of our scheme via numerical simulations, showing that the cloaked object is able to sense the outside world while causing negligible disturbation to the original incident field. Moreover, the cloaking effect is robust against the deviation of the material parameters of cloak from the perfect values. The findings may significantly facilitate the experimental realization of acoustic cloaks and shows many potential applications in a variety of practical situations such as high-precision acoustic measurements in which a reduction of the disturbance caused by acoustic sensor is highly desired.

## Methods

The optimized structure parameters of our dual-layer cloaks are obtained with genetic algorithm (GA). The algorithm selects the fittest individuals and performs the operations of crossover and mutation to create the next generation. The GA begins with the initial population size being 50 and other various parameters such as mutation rate being 0.2. The initial population is first generated and for every following generation, the individuals are ranked based on the fitness scores to find the survivors to mate for creating the next generation. This process would be repeated until the fitness scores satisfy the stopping criteria.

Numerical simulations are performed by COMSOL Multiphysics software. The background medium is chosen as water, for which the mass density and sound speed are 998 kg/m^3^ and 1480 m/s respectively. The structural parameters yielded by the optimization method are used in the simulations for the cloaking media. Perfectly matched layers are used to eliminate the reflected waves by the outer boundaries.

## Additional Information

**How to cite this article**: Gao, H. *et al*. Non-blind acoustic invisibility by dual layers of homogeneous single-negative media. *Sci. Rep.* **7**, 42533; doi: 10.1038/srep42533 (2017).

**Publisher's note:** Springer Nature remains neutral with regard to jurisdictional claims in published maps and institutional affiliations.
